# Matrix metalloproteinase-8 deficiency increases joint inflammation and bone erosion in the K/BxN serum-transfer arthritis model

**DOI:** 10.1186/ar3211

**Published:** 2010-12-29

**Authors:** Samuel García, Jerónimo Forteza, Carlos López-Otin, Juan J Gómez-Reino, Antonio González, Carmen Conde

**Affiliations:** 1 Research Laboratory and Rheumatology Unit, Complexo Hospitalario Universitario de Santiago de Compostela (CHUS), SERGAS, Biomedical Research Institute (IDIS), Travesia da Choupana s/n, Santiago de Compostela, 15706 A Coruña, Spain; 2 Department of Pathology, Complexo Hospitalario Universitario de Santiago de Compostela (CHUS), SERGAS, Biomedical Research Institute (IDIS), Travesia da Choupana s/n, Santiago de Compostela, 15706 A Coruña, Spain; 3 Department of Biochemistry and Molecular Biology, Instituto Universitario de Oncología, Universidad de Oviedo, 33006 Oviedo, Spain; 4 Department of Medicine. University of Santiago de Compostela. San Francisco s/n, Santiago de Compostela, 15782 A Coruña, Spain

## Abstract

**Introduction:**

Rheumatoid arthritis is an autoimmune disease in which joint inflammation leads to progressive cartilage and bone erosion. Matrix metalloproteinases (MMPs) implicated in homeostasis of the extracellular matrix play a central role in cartilage degradation. However, the role of specific MMPs in arthritis pathogenesis is largely unknown. The aim of the present study was to investigate the role of Mmp-8 (collagenase-2) in an arthritis model.

**Methods:**

Arthritis was induced in *Mmp8*-deficient and wildtype mice by K/BxN serum transfer. Arthritis severity was measured by a clinical index and ankle sections were scored for synovial inflammation, cartilage damage and bone erosion. cDNA microarray analysis, real-time PCR and western blot were performed to identify differential changes in gene expression between mice lacking *Mmp8 *and controls.

**Results:**

*Mmp8 *deficiency increased the severity of arthritis, although the incidence of disease was similar in control and deficient mice. Increased clinical score was associated with exacerbated synovial inflammation and bone erosion. We also found that the absence of *Mmp8 *led to increased expression of *IL-1β*, pentraxin-3 (*PTX3*) and prokineticin receptor 2 (*PROKR2*) in arthritic mice joints.

**Conclusions:**

Lack of Mmp-8 is accompanied by exacerbated synovial inflammation and bone erosion in the K/BxN serum-transfer arthritis model, indicating that this Mmp has a protective role in arthritis.

## Introduction

Rheumatoid arthritis (RA) is a chronic autoimmune disease characterized by joint inflammation and progressive destruction of cartilage and bone. Current knowledge of joint destruction indicates that matrix metalloproteinases (MMPs) have a pivotal role in cartilage damage. Articular cartilage is composed of the extracellular matrix and a small number of chondrocytes. Aggrecan and fibrillar type II collagen are the main components of the cartilage extracellular matrix. In RA, depletion of proteoglycans and the subsequent degradation of collagen lead to destruction of articular cartilage. The metalloproteinases induced by IL-1β, TNF, IL-17 and IL-18 are pivotal in this process [1-4].

Multiple pieces of evidence support the relevance of MMPs in the pathogenesis of RA. Several MMPs are highly expressed in the synovial lining and sublining of RA patients and high levels of these proteins have been detected in their sera and synovial fluid [5-7]. Specifically, the high serum levels of MMP-1 and MMP-3 have been proposed as predictors of joint destruction [8]. The role of a few of the MMPs has been analyzed in experimental arthritis models using deficient mice, and the results were variable depending on the MMP analyzed. The effect of Mmp-2 was analyzed in an antibody-induced arthritis model [9]. The *Mmp2*-deficient mice showed significantly exacerbated arthritis compared with wildtype mice, suggesting a suppressive role of Mmp-2 in this model. In contrast, the absence of Mmp-9 was associated with reduced severity of arthritis, indicating the need of Mmp-9 for the development of arthritis [9]. The role of Mmp-3 was analyzed in antigen-induced arthritis and collagen-induced arthritis models [10,11], and a similar incidence and severity of arthritis was displayed by *Mmp3*-deficient and control mice in both arthritis models. This range of results indicates the need to investigate the specific role of individual MMPs in the pathogenesis of RA to identify specific targets.

MMP-8 (collagenase-2) is mainly produced by neutrophils, although it is also expressed by a wide range of cells including chondrocytes [12] and synovial fibroblasts [13]. MMP-8 is a potent collagenolytic enzyme that is involved in the pathogenesis of several inflammatory conditions. Van Lint and colleagues showed that *Mmp8*-deficient mice were protected against TNF-induced lethal hepatitis [14]. Livers of knockout mice did not show the massive influx of neutrophils seen in wildtype mice, probably due to the functional link between Mmp-8 and lipopolysaccharide-induced CXC chemokine, a PMN chemokine. Their work suggests that Mmp-8 is involved in lipopolysaccharide-induced CXC chemokine release and, in turn, in neutrophil recruitment during inflammation. Likewise, the pivotal role of MMP-8 in lipopolysaccharide-induced CXC chemokine, CXCL5 and CXCL8 activation was recently reported [15]. An increased neutrophil accumulation was found, however, in induced skin carcinomas and during wound healing in mice lacking *Mmp8 *[16]. Also, *Mmp8*-deficient mice developed more severe inflammation than wildtype mice in an allergen-induced airway inflammation model and showed more neutrophils in the bronchoalveolar lavage fluid [17]. Overall, these studies indicate that the role of MMP-8 in the inflammatory process is complex and difficult to predict in advance, probably due to specific features of the tissue and stimulus involved in each situation.

Several findings suggest that MMP-8 has a role in RA pathogenesis. It is expressed in serum and synovial fluid from patients with RA. Fibroblast-like synoviocyte cultures from RA patients produce MMP-8 after TNFα stimulation [6,13]. In addition, MMP-8 regulates the activity of several chemokines implicated in RA [18,19]. In the present study we have therefore investigated the impact of *Mmp8 *deficiency in the induced arthritis using the K/BxN serum transfer model. We have also performed a cDNA microarray analysis to investigate differences in the transcriptional profiles from *Mmp8*-deficient and wildtype mice. According to our data, we conclude that Mmp-8 has a protective role in arthritis derived from the ability of this metalloprotease to induce changes in a series of inflammatory mediators.

## Materials and methods

### Mice

Mice lacking *Mmp8 *have been previously described [19] and the KRN T-cell-receptor transgenic mice were a kind gift from C Benoist and D Mathis (Harvard Medical School, Boston, MA, USA; and IGBMC, Strasbourg, France). NOD and C57BL/6 mice were purchased from Charles River (Barcelona, Spain).

*Mmp8*^+/- ^(mixed C57BL/6 × 129Sv background) mice were backcrossed into the C57BL/6 background for 12 breedings. *Mmp8*^+/- ^mice were then intercrossed to generate the *Mmp8*^-/-^, *Mmp8*^+/- ^and *Mmp8*^+/+ ^mice used for arthritis induction.

Genotypes were assessed by PCR of tail DNA. PCR reactions were made using DNA 100 ng, dNTPS 200 μM (Roche, Mannheim, Germany), specific primers 0.5 μM, MgCl_2 _1.5 mM, PCR buffer reaction (Roche) and Taq DNA polymerase 0.5 U (Roche). Amplification was performed using the following conditions: 94°C for 2 minutes, 30 cycles at 94°C for 1 minute, 60°C for 1 minute and 72°C for 1 minute, and a final cycle of elongation at 72°C for 4 minutes. The size of the amplified DNA products was determined in a 1.5% agarose gel in Tris-acetate-EDTA. Amplification of the wildtype allele was carried out with the primer pairs 5'-GTGGATGAATCCCCAGACTC-3' (forward) and 5'-CAAGCAATCAATTCCGGTCT-3' (reverse) [EMBL: AK089234]; and for amplification of the knockout allele, the primers 5'-GTGGATGAATCCCCAGACTC-3' [EMBL: AK089234] (forward) and 5'-TCGCCTTCTTGACGAGTTCT-3' (reverse) [GenBank: DQ890917.2] were used.

K/BxN mice that spontaneously develop arthritis were generated by crossing KRN T-cell-transgenic mice with NOD mice, as previously described [20].

Mice were maintained in the conventional mouse facility of the Medical School of the University of Santiago de Compostela. Animal care was in compliance with Spanish regulations on the protection of animals used for experimental and other scientific purposes (Real Decreto 223/1998). The experimental protocols were approved by the Animal Care and Use Committee of the University of Santiago de Compostela.

### Generation of serum-transferred arthritis and clinical scoring

K/BxN serum was collected from 4-week-old to 8-week-old arthritic K/BxN mice. The serum samples were pooled and stored at -80°C until use. Arthritis was induced by transfer of this pool of sera in 6-week-old to 8-week-old mice in three different experimental groups.

In Group 1, arthritis was induced in 10 *Mmp8*^-/- ^mice, 10 *Mmp8*^+/+ ^mice, and 10 *Mmp8*^+/- ^mice by intraperitoneal injection of 200 μl K/BxN serum on days 0 and 2. These mice were killed on day 14 after serum transfer.

In Group 2, arthritis was induced in 17 *Mmp8*^-/- ^mice and 17 control mice (*Mmp8*^+/+ ^and *Mmp8*^+/-^) by intraperitoneal injection on days 0 and 2 of 100 μl K/BxN serum. These mice were killed for histological assessment on day 9 after serum transfer.

In Group 3, arthritis was induced in nine *Mmp8*^-/- ^male mice and nine *Mmp8*^+/+ ^male mice by injection on days 0 and 2 of 150 μl K/BxN mice serum. These mice were killed for RNA and protein isolation on day 7 after serum transfer.

Arthritis was assessed in each of the four limbs every other day by two blinded observers, using a semiquantitative clinical score (0 = no swelling; 1 = slight swelling and erythema of the ankle, wrist or digits; 2 = moderate swelling and erythema; 3 = severe swelling and erythema; and 4 = maximal inflammation with joint rigidity). The maximum possible score was 16 per mouse.

### Histological analysis

Hind limbs were prepared for histology by dissecting the skin and muscle, and then sectioning ankle joints. Specimens were fixed for 24 hours and demineralized in PBS-0.5 M ethylenediamine tetraacetic acid for 10 days. Ankle joints were embedded in paraffin and sections were cut and stained with hematoxylin and eosin for evaluation of inflammation and bone erosion, as previously described [21]. For analysis of the damage in cartilage, ankle sections were stained with Toluidine blue and Safranin-O following the standard methodology. To determinate osteoclast activity, staining for tartrate-resistant acid phosphatase (TRAP) was performed using the Acid Phosphatase, Leukocyte (TRAP) kit (Sigma, St Louis, MO, USA) following the manufacturer's instructions. Synovial inflammation was scored as previously described [21]: 0 = no inflammation; 1 = slight thickening of synovial cell layer and/or some inflammatory cells in the sublining; 2 = thickening of synovial lining and moderate infiltration of the sublining; 3 = thickening of synovial lining and marked infiltration; and 4 = thickening of synovial lining and severe infiltration.

Cartilage damage was evaluated following a 0 to 4 scale, as previously described [21]: 0 = normal cartilage; 1 = cartilage surface irregularities and loss of metachromasia adjacent to superficial chondrocytes; 2 = fibrillation of cartilage with minor loss of surface cartilage; 3 = moderate cartilage abnormalities including loss of superficial cartilage and moderate multifocal chondrocyte loss; and 4 = marked cartilage destruction with extension of fissures close to subchondral bone.

Bone erosion was scored on a 0 to 4 scale, as previously described [22]: 0 = normal bone; 1 = small areas of resorption; 2 = more numerous areas of resorption; 3 = obvious resorption; and 4 = full-thickness resorption areas in the bone.

Osteoclast activity was evaluated following a scale from 0 to 4 regarding TRAP staining, as previously described [23]: 0 = no staining; 1 = rare positive cells; 2 = some foci of positive cells; 3 = multiple foci; and 4 = diffuse staining. All scores were performed blind with respect to the mouse group.

### Microarray analysis

Total RNA was obtained from ankle joints of three male mice from each of the following groups: *Mmp8*^+/+ ^arthritic mice, *Mmp8*^+/+ ^control mice, *Mmp8*^-/- ^arthritic mice, and *Mmp8*^*-/- *^control mice. Male mice were used because they showed a trend to higher arthritis severity compared with female mice. The joints were taken 7 days after serum transfer and immediately frozen in liquid nitrogen. Subsequent processing was done at Progenika BioPharma SA (Bilbao, Spain).

Total RNA was isolated using the RNeasy Mini Kit and the QIAshredder (Qiagen GmbH, Hilden, Germany) according to the manufacturer's instructions. Integrity of RNA was assessed with the Agilent 2100 Bioanalyzer (Agilent Technologies, Duesseldorf, Germany). Total RNA (300 ng) was subjected to cDNA synthesis and labeling using the Whole Transcrit cDNA synthesis and amplification kit (Affymetrix, Santa Clara, CA, USA). This procedure involves synthesis of cDNA using T7-promoter-containing random primers, which is transcribed subsequently to cRNA. cRNA was quantified and used to generate dUTP-containing cDNA. The enzymes uracyl DNA glycosylase (UDG) and apurinic/apyramidinic endonuclease-1 (APE1) were used to fragment the dUTP-containing cDNA. Complete fragmentation was checked in the Bioanalyzer. Fragmented cDNA was labeled with the terminal transferase-based Whole Transcript Terminal Labeling kit from Affymetrix. Gene expression was evaluated using the Mouse Gene 1.0 ST array (Affymetrix) that contains about 27 probes for hybridization with each of the 28,853 mouse genome transcripts. Quality control procedures recommended by Affymetrix were followed. Intensity raw data were processed following the Robust Multichip Average method. Expression values below background were discarded, leaving information for 18,495 transcripts - of which 11,524 showed variable expression in at least one sample in relation with the others.

### Real-time PCR analysis

Total RNA was obtained from knee joints of six *Mmp8*^+/+ ^and six *Mmp8*^-/- ^male mice 7 days after serum transfer, and from joints of three *Mmp8*^+/+ ^and three *Mmp8*^-/- ^control mice without arthritis, with the RNeasy Kit and RNase-Free DNase Set (Qiagen GmbH) according to the manufacturer's instructions. Total RNA (500 ng) was subjected to cDNA synthesis using the RT^2 ^First Strand Kit (SABiosciences, Frederick, MD, USA). Quantitative real-time PCR was performed in duplicate in a Rotor Gene 6000 thermal cycler (Corbett Research, Cambridge, UK), using the RT^2 ^SYBR Green/Rox qPCR Master Mix (SABiosciences), according to the manufacturer's protocol. The specific primers used in these reactions are listed in Table 1.

**Table 1:** Primer sets used for quantitative PCR study

Gene	Band size (bp)	SABiosciences catalog number	EMBL accession number	Reference position
*IL-1β*	156	PPM03109E	AK168047	1,059 to 1,080
*PROKR2*	136	PPM39370A	AF487279	851 to 870
*PTX3*	99	PMM03342E	BC022176	1,593 to 1,612
*C1QTNF3*	132	PPM37236A	AF246265	811 to 830
*CALPAIN6*	98	PMM27781A	AK145116	2,029 to 2,049
*MMP-3*	94	PMM03673A	AK148467	1,154 to 1,175
*TenascinN*	116	PMM30367A	AF455756	4,477 to 4,499
*β-Actin*	154	PMM02945A	AB028847	163 to 182

Relative levels of gene expression were normalized to the β-actin gene using the comparative Ct method, where Ct is the cycle at which the amplification is initially detected. The relative amount of mRNA from the different genes was calculated using the formula 2^-ΔΔCt, ^where:

$$\Delta \Delta \text{Ct}={\left[{\text{Ct}}_{\text{target}}-{\text{Ct}}_{\text{β-actin}}\right]}_{\text{WT}\;\text{or}\;\text{KO}\;\text{with}\;\text{arthritis}}-{\left[{\text{Ct}}_{\text{target}}-{\text{Ct}}_{\text{β-actin}}\right]}_{\text{WT}\;\text{or}\;\text{KO}\;\text{controls}}$$

For wildtype and *Mmp8*-deficient samples without arthritis, ΔΔCt = 0 and 2^0 ^= 1. For wildtype and knockout samples with arthritis, the value of 2^-ΔΔCt ^indicates the fold change in gene expression relative to the wildtype and knockout controls, respectively. Melting curves and agarose gel electrophoresis were used to assess the amplified band.

### Western blot analysis

Total proteins were obtained from ankle joints of six *Mmp8*^+/+ ^mice and six *Mmp8*^-/- ^mice after 7 days of serum transfer. Whole protein lysates (40 to 50 μg protein) were fractionated by Tris-glycine buffered 10% SDS-PAGE, transferred to Polyvinylidene difluoride membrane (Roche) and probed with antibodies to prokineticin receptor 2 (*PROKR2*) (Santa Cruz, Santa Cruz, CA, USA) and β-actin (Sigma). Bound antibodies were revealed with horseradish peroxidase-conjugated secondary antibodies (Santa Cruz) and the blot developed using a SuperSignal West Femto Maximum Sensitivity Substrate (Pierce, Rockford, IL, USA).

### Statistical analysis

Differences between experimental groups were assessed by repeated-measures analysis of covariance (ANCOVA) and two-sided Mann-Whitney *U *tests. *P *< 0.05 was considered significant. Correlation of histological parameters with clinical scores was determined with the Spearman *R*_S_.

Statistical analysis of the microarray expression results was performed with the Partek Genomics Suite v7.3.1 (Partek, Saint Louis, MO, USA) after normalization with the Robust Multichip Average method and filtering of values below background. Comparisons of expression levels between sample groups were carried out with lineal regression. Significance thresholds were considered applying a False Discovery Rate (FDR) approach or the more conservative Bonferroni correction by the number of independent tests. Functional classification of genes that showed differential expression was done with the DAVID functional annotation clustering utility [24,25]. The default set of 13 gene annotation databases, including three of each of the following functional categories, gene ontology, protein domains and pathways, was used for this clustering. An enrichment score of 3.0 was taken as the threshold for reporting clusters of genes, given that this level corresponds to significant enrichment of the included categories according to a FDR of 0.05. The fold change in expression levels of one group in relation to the other was also obtained after normalization of hybridization signals by the geometric mean of expression levels in all of the arrays.

## Results

### Increased severity of arthritis in mice lacking MMP-8

To ascertain the role of MMP-8 in experimental arthritis, we induced passive K/BxN arthritis in 12-generation B6-backrossed *Mmp8*-deficient (*Mmp8*^-/-^) mice, and their matched wildtype (*Mmp8*^+/+^) and heterozygous (*Mmp8*^+/-^) littermate controls.

In a first experimental group, male and female *Mmp8*^+/+ ^(*n *= 10), *Mmp8*^+/- ^(*n *= 10) and *Mmp8*^-/- ^(*n *= 10) mice were injected intraperitoneally at day 0 and 2 with 200 μl K/BxN mice serum and monitored for signs of arthritis. Evolution of arthritis was evaluated by two blinded observers on a 0 to 4 scale, as described in Materials and methods.

**Figure 1:**
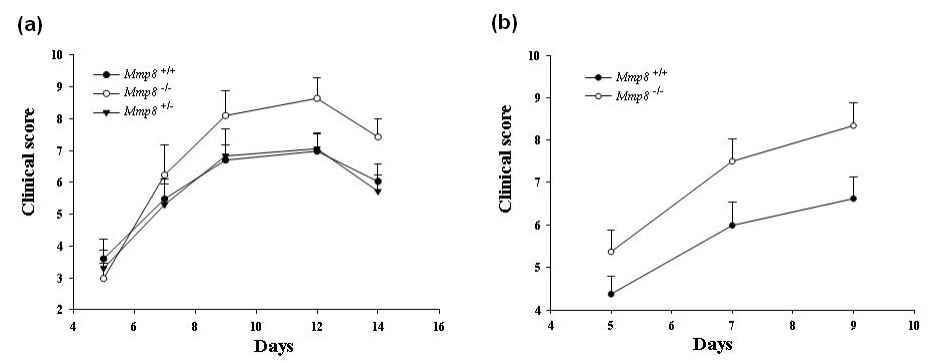
**Increased arthritis severity in *Mmp8*-deficient mice**. **(a) **Clinical score measured in 10 *Mmp8*^-/- ^mice, 10 *Mmp8*^+/+ ^mice and 10 *Mmp8*^+/- ^mice after intraperitoneal injection of 200 μl K/BxN mice serum at days 0 and 2. Values expressed as mean ± standard error of the mean (SEM); *P *= 0.025, by analysis of covariance (ANCOVA) test. **(b) **Clinical score measured in 17 *Mmp8*^-/- ^mice and 17 *Mmp8*^+ ^mice after intraperitoneal injection of 100 μl K/BxN mice serum at days 0 and 2. Values expressed as mean ± SEM; *P *= 0.04, by ANCOVA test.

An incidence of 100% of arthritis was observed in *Mmp8*^-/-^, *Mmp8*^+/+ ^or *Mmp8*^+/- ^mice (Figure 1a). The time course of arthritis was also similar in the three groups of mice. The disease developed rapidly and the maximum of severity was observed between 9 and 12 days. Surprisingly, *Mmp8*-deficient mice displayed significantly higher severity of arthritis than *Mmp8*^+/+ ^and *Mmp8*^+/- ^mice (*P *= 0.025 by repeated-measures one-way ANCOVA test) all through the follow-up. As the severity of arthritis was similar in *Mmp8*^+/+ ^and *Mmp8*^+/- ^mice, these mice were considered a unique control group (*Mmp8*^+^).

To exclude that the system was overloaded by using 200 μl K/BxN serum and to further evaluate the observed differences between *Mmp8 *control and deficient mice, a second experimental group composed of male and female *Mmp8*^-/- ^mice (*n *= 17) and control mice (*n *= 17) was injected intraperitoneally at day 0 and 2 with 100 μl K/BxN serum (Figure 1b). Arthritis was monitored until day 9 and the results confirmed those previously obtained - arthritis severity was significantly higher in *Mmp8*-deficient mice compared with control mice (*P *= 0.04 by repeated-measures one-way ANCOVA test; Figure 1b).

### Increased joint inflammation and bone erosion in *Mmp8*-deficient mice

To quantify joint involvement, we assessed synovial inflammation and bone erosion in hematoxylin and eosin stained sections of ankle joints, and cartilage damage was evaluated in Toluidine blue and Safranin-O stained sections. Right ankles were taken on day 9 after serum transfer from seven *Mmp8*^-/- ^and seven *Mmp8*^+ ^male and female mice of the group injected intraperitoneally with 100 μl K/BxN serum, and a blinded observer scored the histological sections. The clinical score of the *Mmp8*^-/- ^mice was higher than in the *Mmp8*^+ ^mice (*P *= 0.027 by Mann-Whitney *U *test).

**Figure 2:**
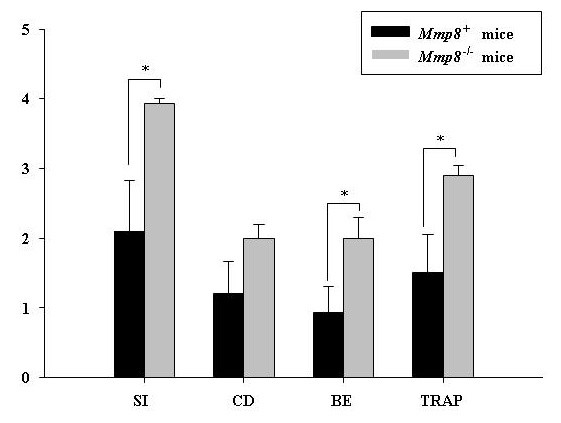
**Increased joint inflammation and bone erosion in mice lacking *Mmp8***. Histologic scores of synovial inflammation (SI), cartilage damage (CD), bone erosion (BE) and tartrate-resistant acid phosphatase (TRAP) staining of ankle sections of *Mmp8*^+ ^mice (*n *= 8) and *Mmp8*^-/- ^mice (*n *= 7) at day 9 after intraperitoneal arthritis induction. Values expressed as mean ± standard error of the mean; *P < 0.05 by two-sided Mann-Whitney *U *test.

**Figure 3:**
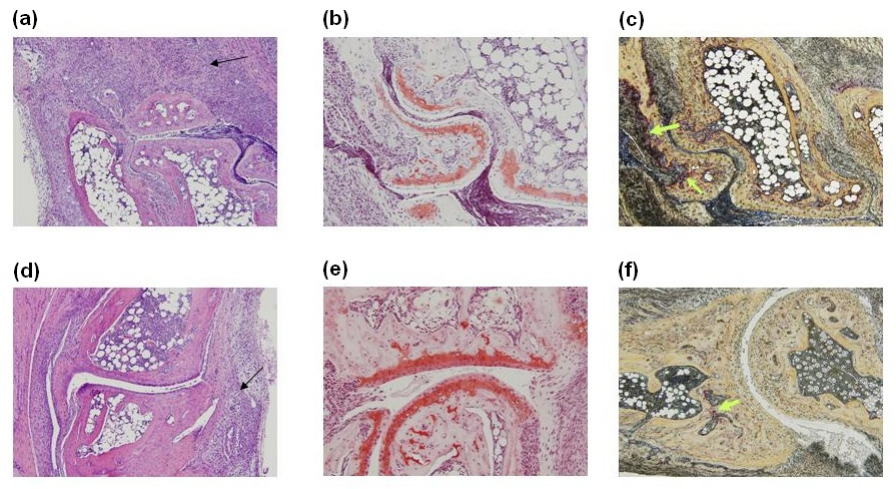
**Higher synovial inflammation and bone erosion in arthritic joints of *Mmp8*-deficient mice than control mice**. Representative sections of ankle joints of *Mmp8*^-/- ^mice **(a)** to **(c)** and *Mmp8*^+ ^mice **(d)** to **(f)** 9 days after intraperitoneal injection of K/BxN serum. More severe inflammation and pannus formation (arrows) were observed on hematoxylin and eosin stained sections from *Mmp8*-deficient mice (a) compared with control mice (d). More severe loss of proteoglycans indicated by destained cartilage was observed on Safranin-O stained sections from *Mmp8*^-/- ^mice (b) compared with *Mmp8*^+ ^mice (e). Increased tartrate-resistant acid phosphatase (TRAP)-positive multinucleated osteoclast-like cells (arrows) were observed at sites of bone erosions on sections stained for TRAP activity from *Mmp8*-deficient mice (c) compared with control mice (f).

Synovial inflammation was scored on a 0 to 4 scale, corresponding to the degree of thickening of the synovial lining and sublining infiltration. A significant increase in synovial inflammation score in *Mmp8*^-/- ^mice compared with *Mmp8*^+ ^mice was observed (*P *= 0.04 by Mann-Whitney *U *test; Figures 2 and 3a,d). Changes in cellular infiltrate composition, however, were not observed in mice lacking *Mmp8 *compared with wildtype mice. Specifically, a similar rate of neutrophils and mononuclear cells were seen in both groups of mice.

As shown in Figures 2 and 3c,f, bone erosion was more marked in *Mmp8*^-/- ^mice than in wildtype mice (*P *= 0.04 by Mann-Whitney *U *test). Furthermore, staining sections for TRAP activity revealed a significantly increase of TRAP-positive multinucleated cells in *Mmp8*^-/- ^mice compared with *Mmp8*^+ ^mice (*P *= 0.025 by Mann-Whitney *U *test). These cells were observed at sites of bone erosion in both groups of mice (Figure 3c,f).

**Figure 4:**
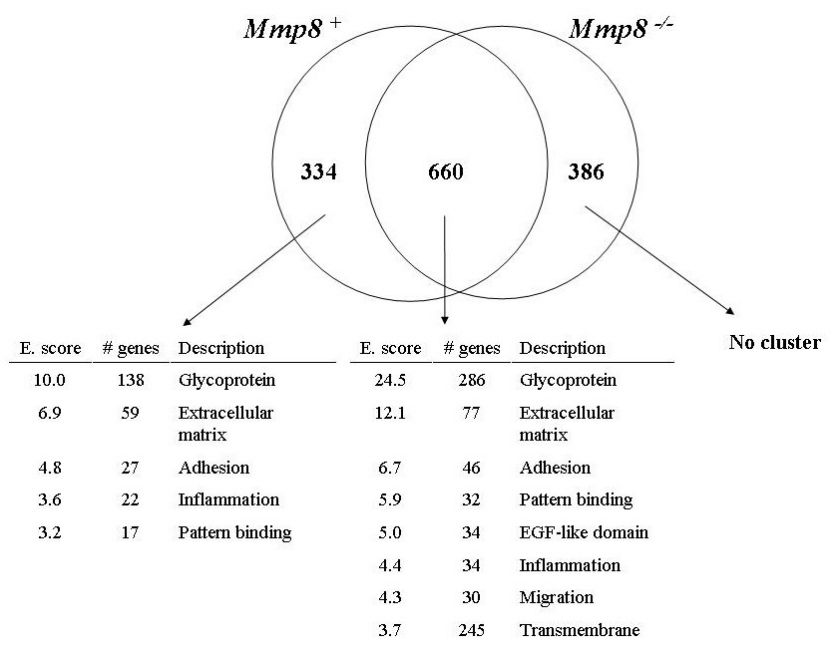
**Clustering of differentially expressed genes**. The DAVID utility [24,25] was used to cluster differentially expressed genes according to a false discovery rate 0.01 threshold, in both *Mmp8*^+/+ ^and *Mmp8*^-/-^arthritic mice, exclusive arthritic *Mmp8*^+/+ ^mice and exclusive arthritic *Mmp8*^-/- ^mice. The functional description and the number of genes with enrichment score (E. score) >3.0 are shown.

A trend to higher cartilage damage in *Mmp8*^-/- ^mice than control *Mmp8*^+ ^mice was detected (Figures 3 and 4b,e), although the difference was not significant (*P *= 0.11 by Mann-Whitney *U *test). Significant correlations between synovial inflammation, cartilage damage, bone erosion and TRAP staining with clinical scores were observed (*R*_S _>0.64 and *P *< 0.017).

Overall these results suggest that MMP-8 plays a protective role in inflammatory arthritis.

### Microarray analysis

To explore the mechanisms underlying the increased arthritis severity in *Mmp8-*deficient mice, we used a genome-wide microarray analysis including probes for more than 28,000 mouse transcripts. Ankle joints from three mice from each of the following groups were studied: *Mmp8*^+/+ ^and *Mmp8*^-/- ^mice with and without arthritis. Mice with arthritis were injected intraperitoneally on days 0 and 2 with K/BxN mice serum, and joints were taken 7 days after injection. Comparison of expression levels between arthritic and nonarthritic control mice yielded a list of about 3,200 genes that were differentially expressed according to an FDR of 5% (2,996 genes in the comparison among *Mmp8*^+/+ ^mice and 3,407 genes in the *Mmp8*^-/- ^comparison), or about 1,000 genes according to the more stringent FDR 1% threshold (Table 2). These lists were largely concordant in the two independent comparisons, as assessed by the fact that most genes differently expressed in *Mmp8*^+/+ ^mice were also differently expressed in *Mmp8*^-/- ^mice. In fact, direct comparison of arthritic *Mmp8*^+/+ ^mice with arthritic *Mmp8*^-/- ^mice did not show any significant difference.

**Table 2:** Number of differentially expressed genes

Mice	Control versus rheumatoid arthritis		
	
	FDR <0.05	FDR <0.01	***P***_**corr **_**<0.05**
*Mmp8*^+/+^	2,996	994	77
*Mmp8*^-/-^	3,407	1,046	52
*Mmp8*^+/+ ^and *Mmp8*^-/-^	2,136	660	40
*Mmp8*^+/+ ^exclusive	860	334	37
*Mmp8*^-/- ^exclusive	1,271	386	12

No differences were found between arthritic *Mmp8*^+/+ ^and arthritic *Mmp8*^-/- ^mice. FDR, false discovery rate.

We therefore conducted other types of analyses. First, we compared the functional groups of differentially expressed genes modified in both groups of arthritic mice, only in arthritic *Mmp8*^+/+ ^mice and only in *Mmp8*^-/- ^mice. The 660 genes that were modified both in *Mmp8*^+/+ ^mice and *Mmp8*^-/- ^mice, according to a FDR 0.01 threshold, could be grouped into eight clusters with an enrichment score over 3.0 (Figure 4). These clusters included some that are more structurally defined and others that are more related with cellular or biological pathways. The same type of analysis was also carried out for the sets of genes that were different only in the *Mmp8*^+/+ ^arthritic mice according to the same FDR 0.01 criteria. There were 334 genes in this class and they were grouped into five clusters of annotations with an enrichment score over 3.0. These five clusters were a subgroup of the eight clusters that were modified in both groups of arthritic mice. The only three clusters missing here were the cluster of epidermal growth factor-like domain proteins and the two last clusters: the one grouping cell migration and motility genes, and the one containing transmembrane proteins. The genes whose expression was significantly modified in arthritic *Mmp8*^+/+ ^mice and not in arthritic *Mmp8*^-/- ^mice were therefore largely from the same functional classes as the genes that were modified in both types of mice.

A similar analysis with the 386 genes that were modified only in arthritic *Mmp8*^-/-^mice gave very different results. No single cluster of genes showed an enrichment score over 3.0, and only two clusters showed a score over 2.0. This indicates that the modified genes specific of arthritis in the *Mmp8*^-/- ^mice are very varied and difficult to group. The pattern of genes that were differentially regulated in *Mmp8*^+/+ ^mice and *Mmp8*^-/- ^mice are therefore very different: the genes regulated specifically in arthritic *Mmp8*^-/- ^mice are similar in number but much more diverse functionally.

The lack of any clearly defined functional class of genes specifically modified in arthritic *Mmp8*^-/- ^mice made it impossible to focus on them to try to discern important factors in the differential arthritis phenotype. We decided to concentrate instead on the genes that, having a most clearly changed expression with arthritis, were also most differentially affected in *Mmp8*^+/+ ^and *Mmp8*^-/- ^mice. We selected the 86 nonredundant genes that were different between arthritic and control mice in the comparison of either *Mmp8*^+/+ ^mice or *Mmp8*^-/- ^mice according to the very conservative Bonferroni-corrected threshold of *P *= 0.05. We obtained the fold change ratios between their respective comparisons. Genes with fold change ratios higher than 1.35 and lower than 0.75 were considered interesting (Table 3). That is, differences between arthritic *Mmp8*^-/- ^mice and their controls were compared with differences between arthritic *Mmp8*^+/+ ^mice and their controls, and the most extreme fold change ratios were selected. Seven out of 29 genes were chosen for confirmatory real-time PCR experiments given their interest in inflammation, autoimmunity or arthritis.

**Table 3:** Genes with the most discordant changes in expression in *Mmp8*^+/+ ^and *Mmp8*^-/-^arthritic mice

Gene	**Fold change: CRL *Mmp8***^**-/- **^**vs. RA *Mmp8***^**-/- **^**(A)**	**Fold change: CRL *Mmp8***^**+/+ **^**vs. RA *Mmp8***^**+/+ **^**(B)**	Ratio A/B
*Anpep*	2.107	3.766	0.559
*Arsi*	3.440	4.685	0.734
*Aspa*	0.289	0.195	1.481
***C1qtnf3***	5.773	8.349	0.691
***Capn6***	5.503	11.324	0.485
*Ces3*	0.050	0.074	0.682
*Csgalnact1*	4.318	5.906	0.731
*Ddefl1*	1.411	2.010	0.702
*Fbln2*	2.254	3.128	0.720
*Gltd5d1*	1.411	2.158	0.654
*Grb10*	1.639	2.421	0.677
*H19*	3.154	5.834	0.541
*Ifitm1*	2.100	4.232	0.496
***Il1*** *β*	5.014	3.711	1.351
*Itga5*	2.184	3.079	0.709
***Mmp3***	27.264	41.104	0.663
*Nt5dc2*	3.532	4.891	0.722
*P4ha3*	6.826	9.504	0.718
*Postn*	2.949	4.571	0.645
***Prokr2***	4.791	3.527	1.358
*Ptgfrn*	1.705	2.407	0.708
***Ptx3***	6.117	4.493	1.361
*Rentla*	0.031	0.043	0.709
*Sfrp1*	8.278	11.900	0.696
*Slc39a14*	5.882	7.949	0.739
*Sult1a1*	0.251	0.181	1.379
*Syne2*	0.590	0.335	1.759
*TenascinN*	3.653	5.793	0.631
*Tpd52*	2.410	1.781	1.353

Genes with the most discordant changes (<0.75 or >1.35) in expression in *Mmp8*^+/+ ^and *Mmp8*^-/-^arthritic mice in relation to their respective nonarthritic controls (selected from arthritis-associated genes according to Bonferroni-corrected *P *< 0.01). Bold genes were chosen for confirmatory real-time PCR. CRL, control; RA, rheumatoid arthritis.

The data discussed in this publication have been deposited in NCBI'S Gene Expression Omnibus [26] and are accessible through the GEO Series accession number [GEO:GSE22971] [27].

### Induction of *IL-1β*, *PROKR2 *and *PTX3 *in arthritic *Mmp8*-deficient mice

**Figure 5:**
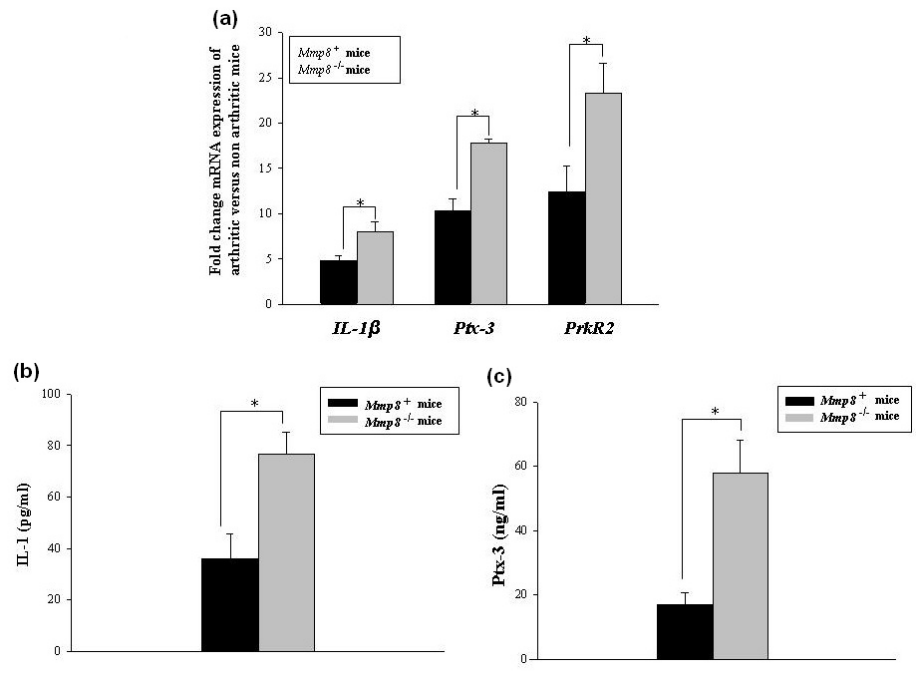
**Increased IL-1β, PTX-3 and PROKR2 mRNA and protein levels in mice lacking *Mmp8***. **(a) ***IL-1β*, pentraxin-3 (*PTX-3*) and prokineticin receptor 2 (*PROKR2*) mRNA levels were measured by quantitative real-time PCR in arthritic joints of *Mmp8*^-/- ^mice (*n *= 6) and *Mmp8*^+/+ ^mice (*n *= 6) at day 7 after intraperitoneal serum injection. Levels of IL-1β **(b) **and PTX-3 **(c) **proteins were measured by ELISA in extracts from arthritic joints of mice at day 7 after intraperitoneal serum injection. Values expressed as mean ± standard error of the mean; *P < 0.05, by two-sided Mann-Whitney *U *test.

To corroborate the results obtained by the microarray analysis, real-time RT-PCR experiments were performed in arthritic joints from six other *Mmp8*-deficient mice and six wildtype mice treated in the same way. Increased mRNA expression of *IL-1β*, *PROKR2 *and pentraxin-3 (*PTX3*) was found in arthritic *Mmp8*^-/- ^mice compared with wildtype mice (*P *= 0.035, *P *= 0.032 and *P *= 0.028, respectively; Figure 5a). Real-time PCR did not, however, confirm the expression changes observed in *CALPAIN 6*, *MMP-3*, *C1QTNF3 *and *TenascinW *in *Mmp8*-deficient mice compared with wildtype mice (data not shown).

**Figure 6:**
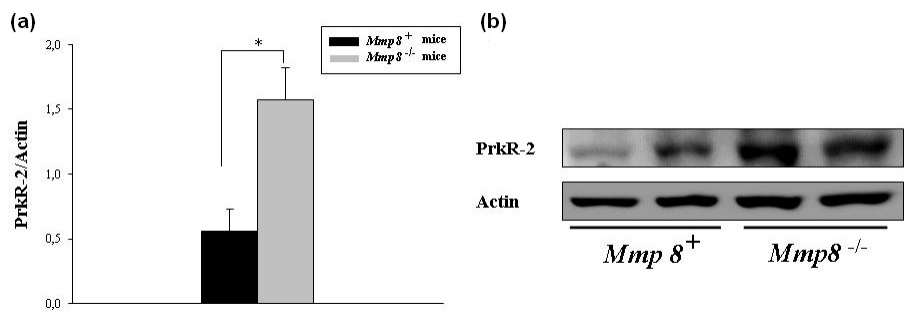
**Increased PROKR2 expression in *Mmp8*-deficient mice**. Protein expression of prokineticin receptor 2 (PROKR2) was determined by western blot in arthritic joints of six *Mmp8*^-/- ^mice and six *Mmp8*^+/+ ^mice at 7 days after arthritis induction. Densitometric analysis of PROKR2 and that normalized with the intensity of actin is shown. **(a) **Data represent mean ± standard error of the mean; *P < 0.05, by Mann-Whitney *U *test. **(b) **Representative blot.

Increased production of *IL-1β *and *PTX3 *was verified by ELISA assay (Figure 5b,c), and results showed a significant increase of both proteins in joints from *Mmp8*^-/- ^mice compared with *Mmp8*^+/+ ^mice (*P *= 0.031 and *P *= 0.017, respectively). PROKR2 production was assessed by western blot and is shown in Figure 6a,b. As expected, PROKR2 levels were significantly higher in joints from *Mmp8*-deficient than in control male mice (*P *= 0.031).

### Discussion

Accumulated evidence indicates that MMPs are involved in the cartilage destruction observed in RA [1-4,7]; MMP inhibitors are thereby of special interest for the treatment of RA. Results from clinical trials of MMP inhibitors in RA have not been encouraging, however, probably due to lack of specificity of such inhibitors [28,29]. In fact, analyses of several MMPs in animal models have shown either [9-11] exacerbation or reduction of arthritis severity depending on the MMP analyzed. This indicates that specific MMPs could have either a promoting or a protective role in arthritis pathogenesis. Knowledge of the role of specific MMPs in the pathogenesis of arthritis therefore seems pivotal to obtain successful inhibitors for treatment. In the present study, we have investigated the impact of lack of *Mmp8 *in the K/BxN serum-transfer arthritis model. The advantages of this transfer model with respect to other arthritis models is its 100% penetrance, early onset, rapid development of osteolytic lesions and its MHC independence. Clinical features and histopathology are very similar to human RA. Another characteristic of the K/BxN transfer model is that it allows us to focus on the effector phase mechanisms of arthritis that are dependent on neutrophils, macrophages, mast cells, and inflammatory mediators, especially IL-1β, but independent of T cells and B cells.

We have found that the absence of *Mmp8 *increased the severity of arthritis without noticeably affecting its time course, either at its onset or at its spontaneous remission. The aggravated clinical course of arthritis was accompanied by exacerbated synovial inflammation and bone erosion. These effects were associated with modified expression of a varied array of genes, including overexpression of *IL-1β*, *PTX3 *and *PROKR2 *in arthritic joints. Surprisingly, despite the known collagenolytic activity of Mmp-8, its absence did not protect from cartilage damage but a trend to increased damage was observed compared with *Mmp8 *wildtype mice. This finding may indicate that other Mmps could compensate for its absence. These data indicate that Mmp-8 plays a protector role against arthritis in this model. This effect is consistent with the previously reported effect of Mmp-8 absence in other inflammation models such as OVA-induced airway inflammation [17], chemically-induced skin carcinomas [19] and skin wound healing [16], in which the absence of Mmp-8 increased the severity of these pathologies and delayed wound healing. In these studies, disease aggravation was linked to increased neutrophil accumulation in the mice lacking *Mmp8*. In our work, we did not observe differences in cellular infiltrate composition between *Mmp8 *control and deficient mice, suggesting that mechanisms involved in the Mmp-8 regulation of inflammation are complex and include its effect in other aspects of inflammation as shown by our expression studies. It is possible that differences between models depend on the nature of the inflammatory stimulus or of the tissue affected.

To elucidate the mechanisms behind arthritis aggravation in *Mmp8*^-/- ^mice, we have investigated the gene expression profile in *Mmp8*-sufficient and *Mmp8*-deficient mice with and without arthritis using microarray technology. There was a wide array of genes that changed expression in arthritic mice. Most were coincident in *Mmp8*-sufficient and *Mmp8-*deficient mice, and they can be grouped in functional categories that are congruent with current knowledge of arthritis mechanisms. The functional spread of the genes whose expression was only modified in the arthritic *Mmp8*^-/- ^mice contrasted with the clustering in five functional categories of the genes significantly modified only in the arthritic *Mmp8 *wildtype mice, despite being similar in number. This result is consistent with the lack of any clearly different phenotype in the histological analysis and has been taken into consideration to interpret the analyses of individual genes. To select genes for detailed analysis, we decided to focus on the genes that with high likelihood were differentially expressed with respect to arthritis and the presence of *Mmp8*. After selection of a group of seven genes, we found an increased expression of *IL-1β*, *PTX3 *and *PROKR2 *in arthritic joints from *Mmp8*-deficient mice compared with wildtype mice that were confirmed by real-time PCR assays. The corresponding increase in protein expression was validated by ELISA and western blot.

*IL-1β *is highly expressed in the synovium of RA patients and plays a crucial role in production of inflammatory mediators and articular damage [2,30]. This cytokine's functional relevance has been demonstrated in several animal models, including the K/BxN model [30-36]. Results of these studies indicate that the increased *IL-1β *expression observed in *Mmp8*-deficient mice can contribute to the higher clinical score, synovial inflammation, osteoclast activity and bone erosion found in these mice.

PTX3 is the prototypic member of the long pentraxin family of acute phase reactants. PTX3 rapidly increases in serum during endotoxic shock, inflammation and infections [37]. A possible role of this protein in potentiating inflammation has been reported in a model of intestinal injury by ischemia/reperfusion in which PTX3 transgenic mice showed exacerbated inflammatory response and increased lethality [38]. Also, mice lacking PTX3 displayed reduced tissue inflammation and increased survival rates [39]. Our results showed an increased PTX3 expression in mice lacking *Mmp8 *compared with wildtype mice, where it was also increased, indicating that PTX3 upregulation could have contributed to the higher arthritis severity in the knockout mice. This result suggests that the accumulation of PTX3 in the synovial fluid of RA patients after being produced by synoviocytes and synovial endothelial cells [40] can be also a contributor to the inflammation process.

PROKR2 is a seven-transmembrane coupled G-protein receptor that binds prokineticin-2. PROKR2 is highly expressed in the bone marrow, and in neutrophils, monocytes and dendritic cells [41]. Signaling through this receptor induces survival, differentiation and activation of granulocytic and monocytic lineages [42]. The higher expression of PROKR2 found in the arthritic joints from *Mmp8*-deficient mice could therefore have contributed to the increased inflammatory infiltration observed in them.

Changes in the expression of these three genes exemplify different ways in which the lack of MMP-8 led to an aggravation of arthritis: promotion of inflammation by *IL-1β *and other molecules like *PTX3*, induction of maturation and activation of osteoclasts by *IL-1β *and *PROKR2*, and enhanced inflammatory infiltrate by *IL-1β *and possibly *PROKR2 *- however, other contributing mechanisms are possible as only a fraction of the genes with possible differential expression were analyzed. Similar analysis in other models of inflammation will help to unravel the many ways in which MMP-8 seems to protect against inflammation.

## Conclusions

The present study indicates that MMP-8 protects against inflammatory synovitis and bone erosion in the K/BxN serum-transfer arthritis model. Expression analysis indicates that protection is due to changes in multiple genes belonging to different functional categories. Three of these genes have been validated exemplifying the involved pathways: *IL-1β*, *PTX3 *and *PROKR2*.

## Abbreviations

ANCOVA: analysis of covariance; MMP: matrix metalloproteinases; ELISA: enzyme-linked immunosorbent assay; FDR: false discovery rate; IL: interleukin; PBS: phosphate-buffered saline; PCR: polymerase chain reaction; RA: rheumatoid arthritis; PTX3: pentraxin-3; PROKR2: prokineticin receptor 2; TRAP: tartrate-resistant acid phosphatase; C1QTNF3: C1q and TNF-related protein; TNF: tumor necrosis factor.

## Competing interests

The authors declare that they have no competing interests.

## Authors' contributions

SG carried out the experiments and participated in the analysis of data and in drafting the manuscript. JF carried out the histological scoring. CL-O participated in design of the study and revision of the manuscript. JJG-R participated in design and coordination of the study, and revision of the manuscript. AG performed the statistical analysis, and participated in design of the study, interpretation of data and drafting the manuscript. CC conceived of the study, and participated in its design, in coordination and interpretation of the data and in drafting the manuscript. All authors read and approved the final manuscript.
